# Sodium Glucose Cotransporter 2 (SGLT2) Plays as a Physiological Glucose Sensor and Regulates Cellular Contractility in Rat Mesangial Cells

**DOI:** 10.1371/journal.pone.0151585

**Published:** 2016-03-21

**Authors:** Masanori Wakisaka, Tetsuhiko Nagao, Mototaka Yoshinari

**Affiliations:** 1 Wakisaka Naika (Clinic of Internal Medicine), Fukuoka City, Japan; 2 Midori no Clinic, Fukuoka City, Japan; 3 Yoshinari Int. Med. Clinic, Fukuoka City, Japan; University of Louisville, UNITED STATES

## Abstract

**Purpose:**

Mesangial cells play an important role in regulating glomerular filtration by altering their cellular tone. We report the presence of a sodium glucose cotransporter (SGLT) in rat mesangial cells. This study in rat mesangial cells aimed to evaluate the expression and role of SGLT2.

**Methods:**

The SGLT2 expression in rat mesangial cells was assessed by Western blotting and reverse transcription-polymerase chain reaction (RT-PCR). Changes in the mesangial cell surface area at different glucose concentrations and the effects of extracellular Na^+^ and Ca^2+^ and of SGLT and Na^+^/Ca^2+^ exchanger (NCX) inhibitors on cellular size were determined. The cellular sizes and the contractile response were examined during a 6-day incubation with high glucose with or without phlorizin, an SGLT inhibitor.

**Results:**

Western blotting revealed an SGLT2 band, and RT-PCR analysis of SGLT2 revealed the predicted 422-bp band in both rat mesangial and renal proximal tubular epithelial cells. The cell surface area changed according to the extracellular glucose concentration. The glucose-induced contraction was abolished by the absence of either extracellular Na^+^ or Ca^2+^ and by SGLT and NCX inhibitors. Under the high glucose condition, the cell size decreased for 2 days and increased afterwards; these cells did not contract in response to angiotensin II, and the SGLT inhibitor restored the abolished contraction.

**Conclusions:**

These data suggest that SGLT2 is expressed in rat mesangial cells, acts as a normal physiological glucose sensor and regulates cellular contractility in rat mesangial cells.

## Introduction

Since the Na^+^/glucose cotransport hypothesis was first proposed, many investigators have examined sodium glucose cotransporters (SGLTs) in the intestine, kidney, brain, and thyroid gland [1]. In 1987, Hediger et al. reported the cloning of SGLT1 [2], and Wright et al. later cloned additional SGLTs. They reported that the SGLT gene family (the SLC5 family) is a large group of proteins with 12 human family members. The SLC5 family encodes 60- to 80-kDa proteins containing 580–718 amino acids [1]. SGLT1 and SGLT2 are the most widely studied glucose cotransporters.

We previously reported the expression of SGLT and facilitated glucose transporter 1 (GLUT1) in rat mesangial cells and bovine retinal pericytes [3–6]. Prior to these reports, SGLT was believed to only localize to intestinal and renal tubular epithelial cells. Epithelial cells in the intestine and the renal late proximal tubules (S3 segment) express SGLT1, whereas cells in the renal proximal tubules in the S1 and S2 segments express SGLT2 [7]. These isoforms differ with respect to their affinity for glucose, their transport capacity for glucose, and the ratio of concomitant Na^+^ and glucose transport [7–9]. Rat mesangial cells and retinal pericytes had almost the same glucose Km values, which were high enough to suggest the expression of SGLT2 [3, 4]. Galactose transport also differs between SGLT1 and SGLT2. SGLT1 transports galactose but SGLT2 does not [8]. SGLT in bovine retinal pericytes does not transport D-galactose, suggesting that the SGLT in bovine retinal pericytes is SGLT2 [6]. Currently, which isoform of SGLT is present in rat mesangial cells is unclear. New anti-diabetic SGLT2 inhibitors blocking glucose reabsorption via SGLT2 in proximal tubular epithelial cells have become available to treat diabetic patients [10]. However, SGLT2 inhibitors may affect all cells that express SGLT2 rather than only renal proximal tubular epithelial cells. It is therefore important to identify the isoform of SGLT in mesangial cells [3].

In early diabetic nephropathy, glomerular hyperfiltration is important, which is primarily explained using the glomerular hemodynamic hypothesis [11] or tubuloglomerular feedback [12]. These mechanisms are based on the balance between glomerular afferent and efferent arteriolar tone in the glomerulus [13]. However, mesangial cells also play important roles in the maintenance and regulation of glomerular microcirculation [14]. In microcirculation, mesangial cells and retinal pericytes regulate the capillary surface area by changing their contractility, which regulates microvascular blood flow and transluminal filtration [15–18]. Mesangial cells are known to lose contractility under high glucose conditions [19, 20], which is hypothesized as one of the causes of glomerular hyperfiltration [21]. In the early stages of retinopathy, dilatation of retinal vessels is observed following retinal pericyte swelling and loss [22–24].

Various substances have been reported to induce contraction of mesangial cells, including angiotensin II, endothelin and serotonin [25–27]. Calcium entry is required for cellular contraction and is achieved primarily via a voltage-sensitive Ca^2+^ channel [28, 29]. However, the Na^+^/Ca^2+^ exchanger (NCX) also plays a role in Ca^2+^ entry and cellular contraction, and its presence has been reported in cardiomyocytes and smooth muscle cells [30, 31]. Mesangial cells have also reported to contain NCX [32, 33]. NCX has two modes of calcium-to-sodium exchange, namely Ca^2+^ exit and Ca^2+^ entry modes, depending on the Na^+^ and Ca^2+^ concentration gradients, with a Na^+^ to Ca^2+^ exchange ratio of almost 3:1 [34]. We have reported that cultured bovine retinal pericytes change their cellular contractility according to the extracellular glucose concentration via SGLT2 and NCX [6]. The Km value of SGLT in mesangial cells appears to be sufficiently high to act as a sensor for blood glucose levels. Based on these findings, we speculate that SGLT may have a major influence on the contractility of mesangial cells, as well as bovine retinal pericytes, via NCX in response to changes in the extracellular glucose concentration [6].

The present study aimed to determine the following: whether SGLT2 is expressed in cultured rat mesangial cells by examining the SGLT2 protein and mRNA expression using Western blotting and RT-PCR; the role of SGLT2 in regulating contractility during exposure to different glucose concentrations in rat mesangial cells; and the extent of cellular swelling and loss of contractile response to angiotensin II under a long-term high glucose condition.

## Methods

### Animal Care and Cell Preparation

Mesangial cells were isolated from the kidneys of four-week-old Sprague-Dawley rats according to a previously reported differential sieving procedure [34]. Cells were cultured on plastic plates (NUNC Brand Products, Denmark) in a 1:1 mixture of Dulbecco's modified Eagle’s medium (DMEM) and Ham's F-12 (both from Sigma-Aldrich, St. Louis, MO) containing 10% fetal bovine serum (FBS) (GIBCO BRL, Grand Island, NY), 10% Nu-serum (Collaborative Research, Bedford, MA), penicillin (100 U/ml), and streptomycin (100 μg/ml) (Sigma-Aldrich). Rat mesangial cells at passages 3–8 were used for the following experiments after reaching greater than 90% confluence because the SGLT expression in rat mesangial cells has been reported to disappear after the 12th passage [3]. Renal proximal tubular epithelial cells from the same rats were prepared as previously described [35]. The study protocol was approved by the Animal Ethics Review Committee of the FUKUOKA SGLT2 Study Group (FSGLTSG-2010-14). All animal care and procedures were performed in accordance with the Guidelines of the Japanese Government Animal Protection and Management Law and the Japanese Government Notification on Feeding and Safekeeping of Animals.

### SGLT2 Western Blotting

A sequence of amino acids from rat SGLT2 (GMSKSGSGSPPP) that is absent from rat SGLT1 [36, 37] was synthesized. IgG antibodies against rat SGLT2 were obtained by immunizing a Kb1:JW rabbit with the synthesized peptide antigen. To perform Western blotting for SGLT2, membrane proteins were extracted as previously reported [35]. Briefly, rat mesangial cells and renal proximal tubular epithelial cells were sonicated in a buffer containing 0.25 M sucrose, 5 mM sodium diatrizoate, 100 mM phenylmethylsulfonyl fluoride, and 10 mM NaHCO_3_ (pH 7.0). After centrifugation at 1,200 x *g* for 10 min, the supernatant was centrifuged again at 9,000 x *g* for 1 hr. The supernatant was then centrifuged at 190,000 x *g* for 1 hr. The pellet was resuspended in the same buffer, applied to a discontinuous sucrose gradient buffer (25%, 30%, and 35%), and then centrifuged at 150,000 x *g* for 16 hr. The membrane fraction in 25% sucrose was collected and centrifuged at 190,000 x *g* for 1 hr after 10-fold dilution with buffer. The pellet was then collected for Western blotting. Protein extracts (50 μg) from mesangial cells and a positive control membrane protein from renal proximal tubular epithelial cells were separated on 10% acrylamide gels overlaid with 3.5% stacking gels by sodium dodecyl sulfate polyacrylamide gel electrophoresis and then transferred to nitrocellulose membranes. The membranes were incubated with a rabbit IgG antibody against SGLT2 (1:1,000 dilution) followed by peroxidase-linked protein A (Amersham). Immunoreactive bands were visualized using ECL reagents (Amersham) and X-Omatic AR film (Eastman-Kodak, Rochester, NY).

### Reverse Transcription-Polymerase Chain Reaction (RT-PCR)

Total RNA was prepared from rat mesangial cells and renal proximal tubular epithelial cells using Isogen (Wako Junyaku, Osaka, Japan). The following SGLT2 PCR primers were synthesized based on the reported DNA sequence of SGLT2: forward primer sequence (5' CCTGCTGCGTGACCCTGTGA 3') and reverse primer sequence (5' AGAGTGTGCTGCTGCTGTTA 3') [36–38]. RT-PCR was performed using 1 ng of total RNA from rat mesangial cells, smooth muscle cells, or renal proximal tubular epithelial cells, an Oligo(dT) 15 primer (Promega, Madison, WI), 0.5 μM of each primer, and Ready-To-Go RT-PCR Beads (Amersham Pharmacia Biotech Inc., NJ). Reverse transcription to cDNA was performed at 42°C for 30 min. The following PCR amplification program was used on a thermal cycler (model 9600, Perkin Elmer-Cetus, Foster City, CA): 35 cycles of 30 sec at 95°C, 30 sec at 55°C, and 1 min at 72°C.

### Cellular Contraction Experiment

Changes in the tonus of mesangial cells were examined through surface area measurements, as previously reported [6]. Changes in the cell surface area after incubation with conditioned media for 60 min at 37°C were recorded every 20 min using a light microscope equipped with a digital camera (Nikon, Tokyo, Japan). Serial changes in the cell surface area were measured using NIH Image (written by Wayne Rasband, National Institutes of Health, Bethesda, MD). The cells were incubated in 20 mM Tris/HEPES (pH 7.4) buffer containing 5 mM KCl, 2.5 mM MgSO_4_ and 1 mM CaCl_2_ with different concentrations of glucose (2.5, 5, 10 and 20 mM) in the presence (145 mM NaCl) or absence (145 mM choline-Cl) of sodium after three washes using the same buffer. To determine the effect of extracellular calcium ions, cells were incubated in 20 mM Tris/HEPES (pH 7.4) buffer containing 5 mM KCl, 2.5 mM MgSO_4_ and 2 mM EGTA without CaCl_2_. The cells were also incubated with an SGLT inhibitor, 10^-7^M phlorizin (Sigma), inhibitors of the Na^+^/Ca^2+^ exchanger, 5 x 10^-5^ M 2’,4’-dichlorobenzamil-HCl or 5 x 10^-5^ M benzamil-HCl (Molecular Probes, Eugene, OR), and a voltage-sensitive calcium channel inhibitor. In addition, 10^-7^ M nicardipine (Sigma) was also added to 20 mM Tris/HEPES buffer (pH 7.4) containing 1 mM CaCl_2_, 5 mM KCl and 2.5 mM MgSO_4_ with 5 mM or 30 mM glucose in the presence of sodium. We also tested the effects of non-metabolizable sugars, 25 mM α-methyl-glucoside (AMG) and 25 mM 2-deoxy-glucose (2DOG), and angiotensin II (Bachem, Bubendorf, Switzerland) on the contraction of mesangial cells.

### Effect of a Long-term High Glucose Condition on Cellular Size and Contractile Response in the Presence and Absence of Phlorizin

Mesangial cells were incubated with 5 mM and 20 mM glucose with or without phlorizin for 6 days. The cellular size was measured as in our previous report [5]. Briefly, confluent rat mesangial cells were incubated in DMEM containing 5% FBS and 5% Nu-serum with either 5 or 20 mM glucose with and without 10^-7^ M phlorizin on 6-well plates (Nunc) for 6 days. The media were changed every day to maintain the glucose concentration in the media. After the cells were washed three times with PBS, they were treated with PBS containing trypsin (Sigma) for 2 min to separate them to single cells. Next, the cells were incubated for 30 min at 37°C in the Tris/HEPES buffer (pH 7.4) with 5 mM glucose and an osmolarity of 280 mOsm in an atmosphere of 95% air and 5% CO_2_. The diameters of all single cells under these conditions were measured using light-microscopy photographs.

### Statistical Analysis

The data are presented as the mean ± SD. Differences between groups were examined for statistical significance using one-way analysis of variance (ANOVA), with the Bonferroni multiple comparisons post hoc test. A p value less than 0.05 denoted statistical significance.

## Results

**Fig 1:**
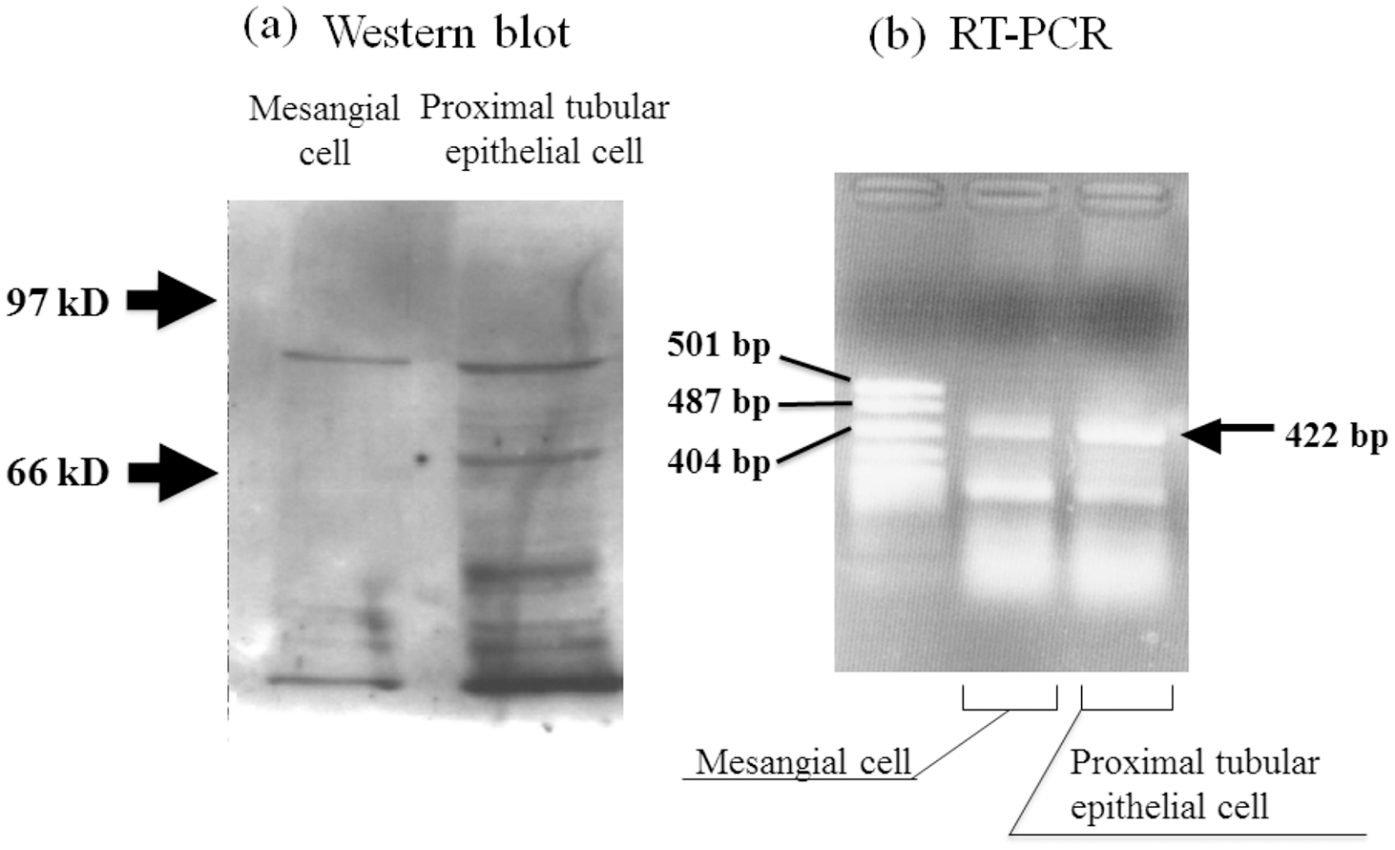
Western blot (a) and RT-PCR (b) of sodium glucose cotransporter 2. The IgG antibody reacted with membrane proteins of the appropriate predicted molecular weight (73 kDa) from both mesangial cells (left) and renal proximal tubular epithelial cells (right). RT-PCR products for SGLT2 from the total RNA from both mesangial cells (A: left) and renal proximal tubular epithelial cells (B: right) revealed a single band of the appropriate predicted size (422 bp).

To identify the SGLT isoforms in rat mesangial cells, Western blotting for SGLT2 was performed. A Western blot analysis yielded a 73-kDa band that reacted with the rabbit anti-SGLT2 IgG antibody (Fig 1a). The antibody reacted with a membrane protein in rat mesangial cells and in renal proximal tubular epithelial cells. The SGLT2 antibody did not react with the membrane proteins of rat smooth muscle cells, which do not perform sodium-dependent glucose uptake (data not shown). A band of the predicted 422-bp size was detected among the RT-PCR products from the total mRNA from rat mesangial cells and renal proximal tubular epithelial cells, consistent with SGLT2 mRNA expression in rat mesangial cells (Fig 1b).

**Fig 2:**
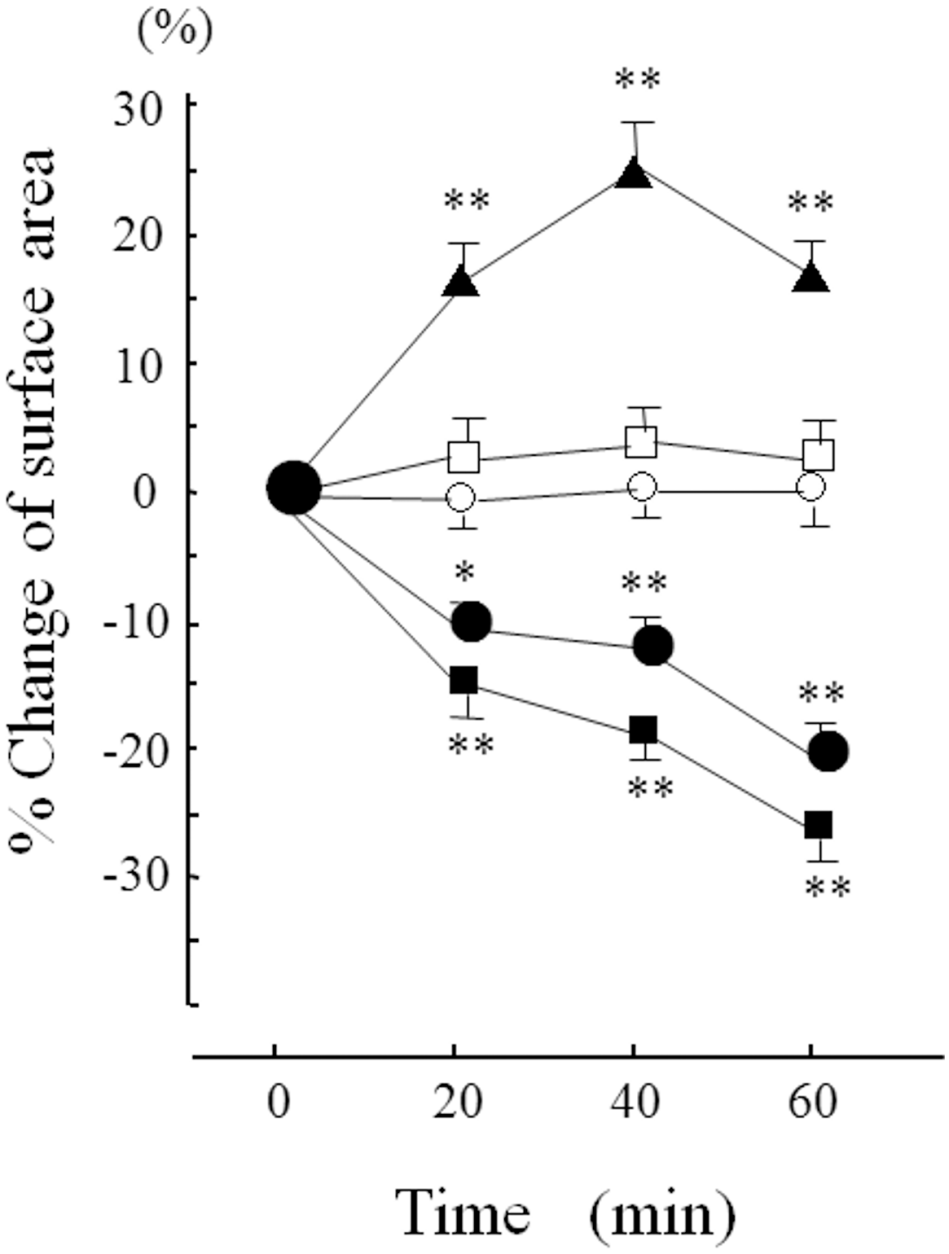
Serial changes in the cell surface area at different glucose concentrations. ▲: 2.5 mM glucose, ○: 5 mM glucose, □: 5 mM glucose + mannitol, ●: 10 mM glucose, ■: 20 mM glucose. n = 6 in each condition. Data are the mean ± SD. Changes in surface area were measured every 20 min. *: p<0.05 *vs* 5 mM glucose for the same period, **: p<0.01 *vs* 5 mM glucose for the same period.

Serial changes in the surface area of rat mesangial cells exposed to different glucose concentrations are shown in Fig 2. The cell surface area did not change at 5 mM glucose. Higher glucose concentrations in the buffer were associated with a reduction in the surface area in a concentration-dependent manner, and 20 min of these conditions significantly decreased the area compared with those of cells in 5 mM glucose. Conversely, the cell surface area increased in a glucose concentration of 2.5 mM, and the increase was significant after 20 min. However, an osmolality equivalent to 20 mM glucose, created by adding 15 mM mannitol to 5 mM glucose, had no effect on the cellular size.

**Fig 3:**
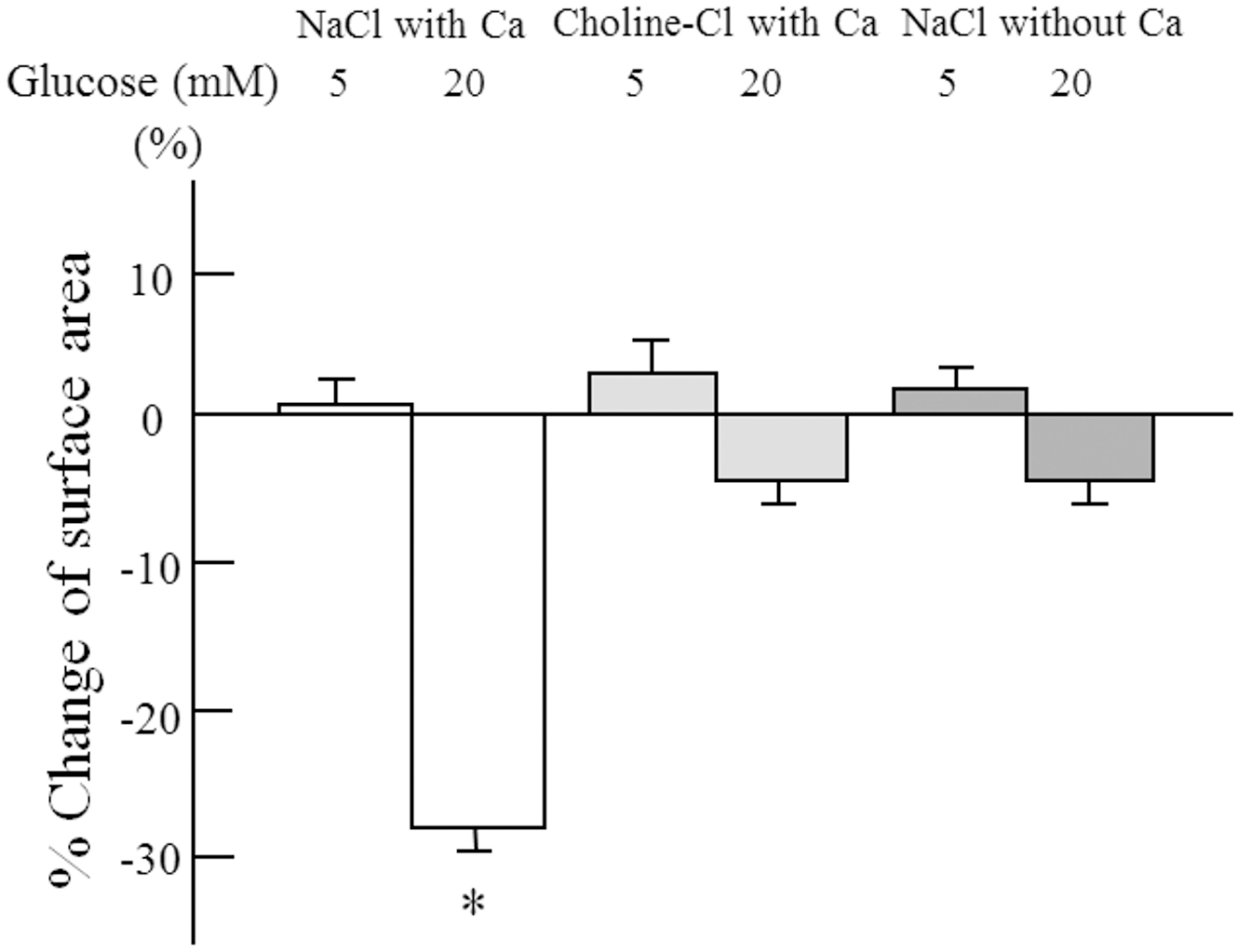
Effects of extracellular sodium and calcium on cellular contraction. Cell surface area was determined after a 60 min incubation in the presence (NaCl) and absence (choline-Cl) of sodium and calcium. n = 6 in each condition. Data are the mean ± SD. *: p<0.001 *vs* 5 mM glucose.

The effect of high glucose concentrations on the cell surface area was observed only in the presence of both extracellular Na^+^ and Ca^2+^ (Fig 3). No reduction in surface area was observed in the absence of either extracellular Na^+^ or Ca^2+^, even under high glucose conditions.

**Fig 4:**
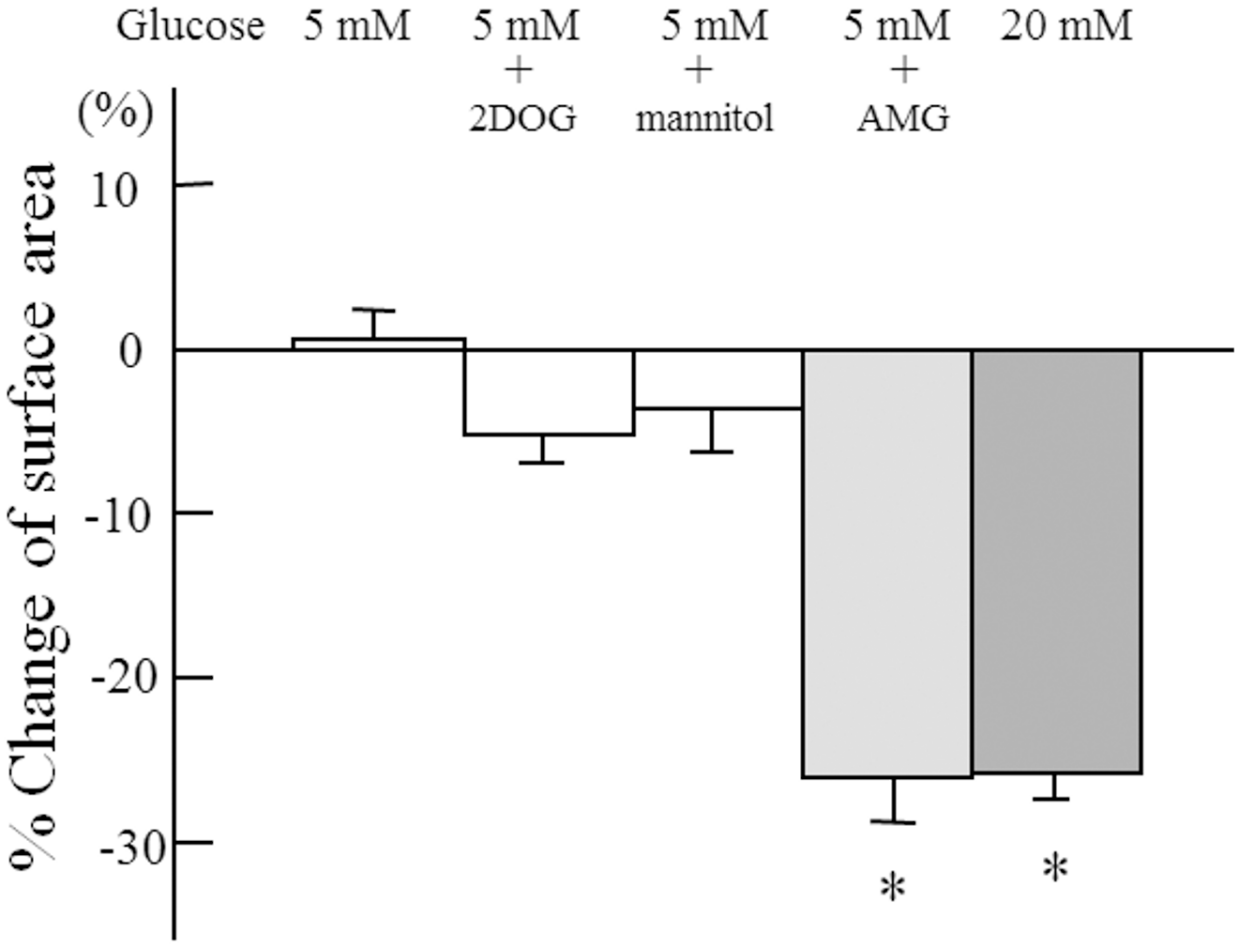
Effects of non-metabolizable sugars on cellular contraction. Cell surface area was determined after exposure for 60 min to conditioned media. AMG: 15 mM α-methyl-glucoside, mannitol: 15 mM mannitol, 2DOG: 15 mM 2-deoxy-glucose. n = 6 in each condition. Data are the mean ± SD. *: p<0.005 *vs* 5 mM glucose.

The effect of non-metabolizable sugars on the cell surface area was tested, including AMG, which enters the cell via SGLT2, and 2DOG, which enters through a facilitated glucose transporter (Fig 4). The cell surface area decreased only in the presence of AMG, whereas the area did not change in the presence of 2DOG or mannitol.

**Fig 5:**
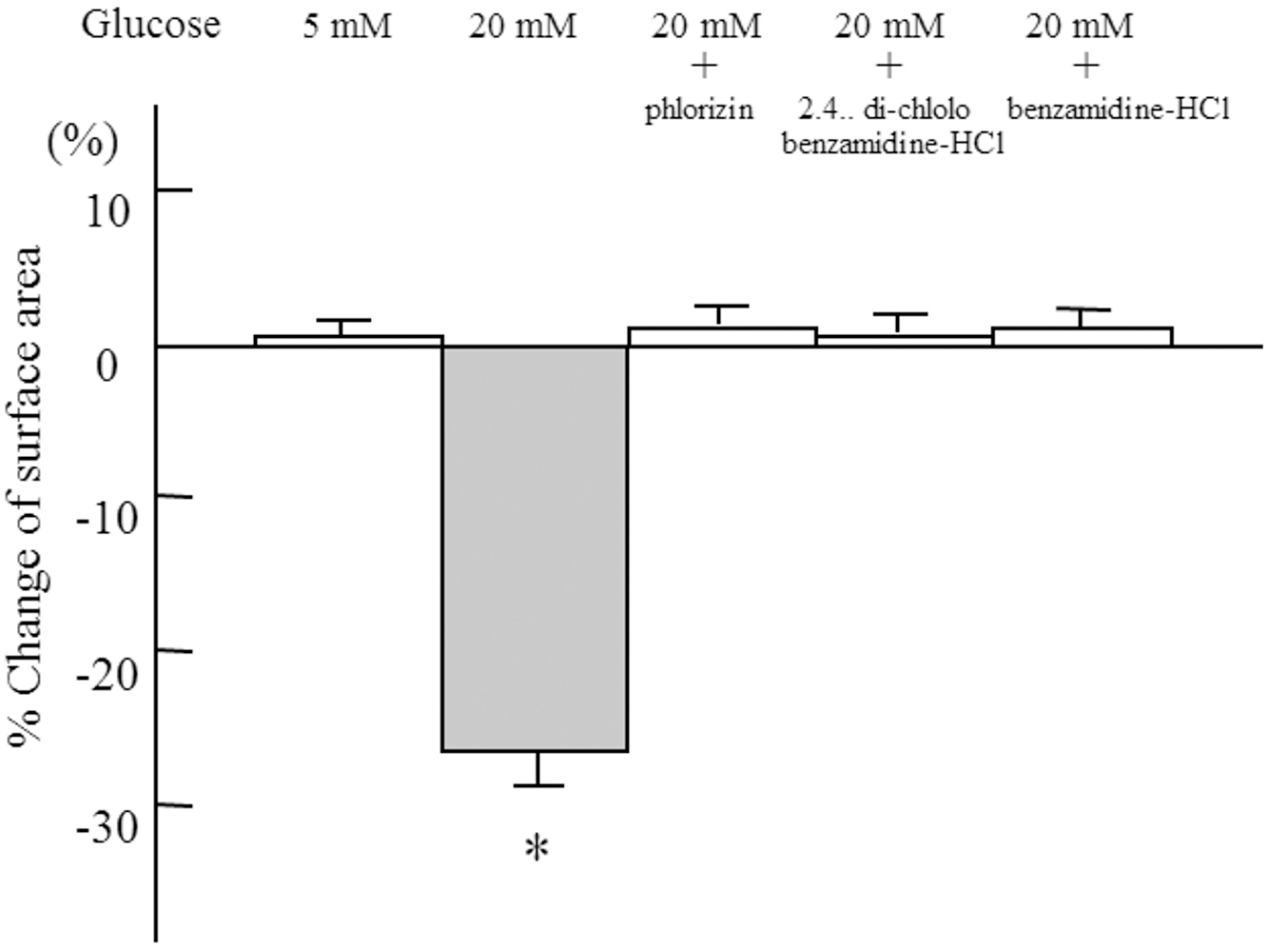
Inhibitory effect of the sodium-coupled glucose transporter and the Na^+^/Ca^2+^ exchanger on glucose-induced cellular contraction. Cell surface area was determined after 60 min of exposure to conditioned media. n = 6 in each condition. Data are the mean ± SD. *: p<0.001 *vs* 5 mM glucose.

Fig 5 shows the inhibitory effect of phlorizin, an inhibitor of SGLT, and 2’,4’-dichlorobenzamil-HCl and benzamil-HCl, inhibitors of NCX, on cellular contraction. These inhibitors abolished the high glucose-induced cellular contraction.

**Fig 6:**
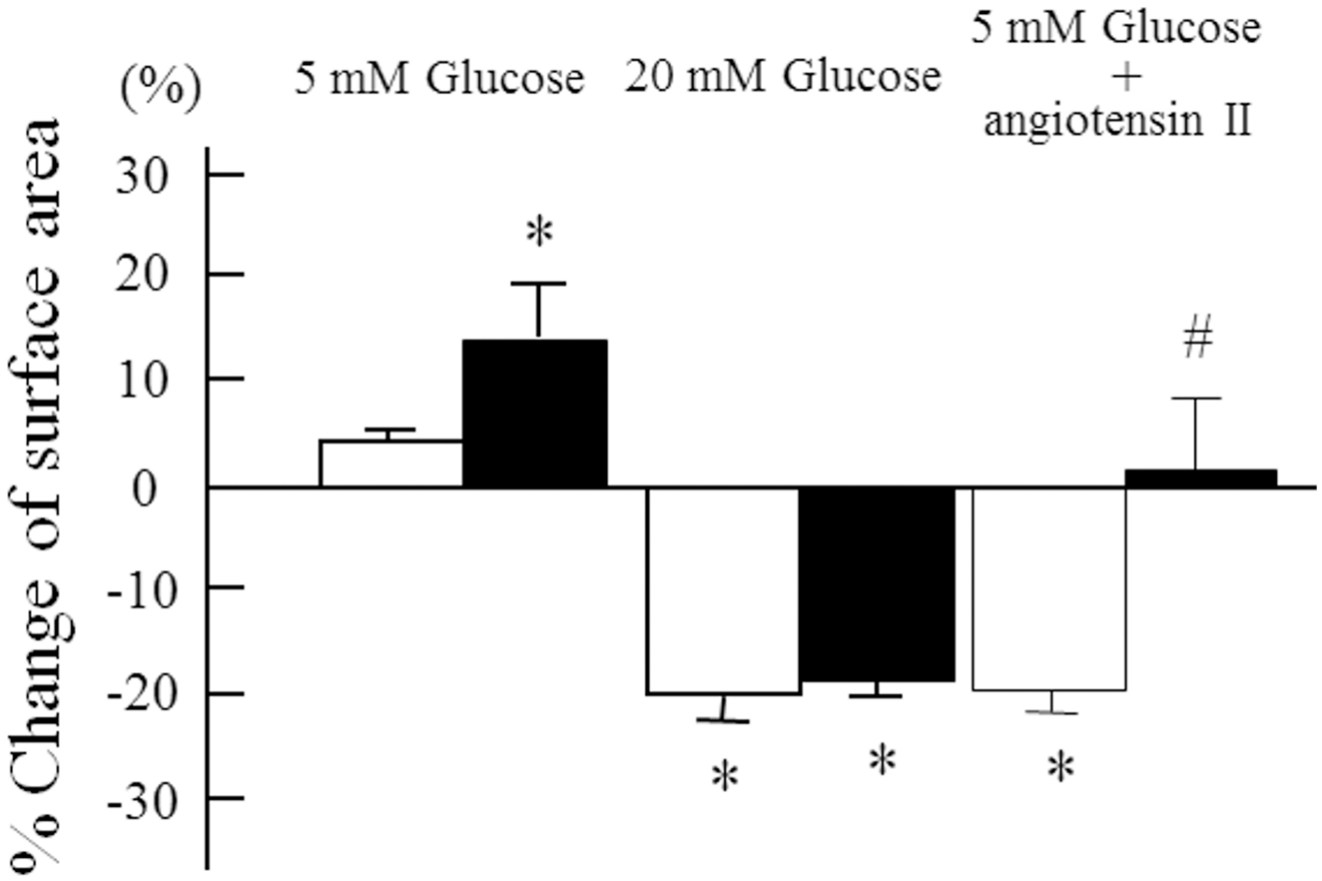
Effects of a calcium channel blocker on glucose-induced cellular contraction. Cell surface area was determined after 60 min of exposure to conditioned media with either 5 mM glucose, 20 mM glucose or 10^-7^ M angiotensin II in the presence (□) or absence (■) of nicardipine. n = 6 in each condition. Data are the mean ± SD. *: p<0.05 *vs* 5 mM glucose. #: p<0.001 *vs* 20 mM glucose with and without nicardipine.

However, nicardipine, a calcium channel blocker, had no effect on high glucose-induced cellular contraction but did increase the surface area in 5 mM glucose and inhibited angiotensin II-induced cellular contraction (Fig 6).

**Fig 7:**
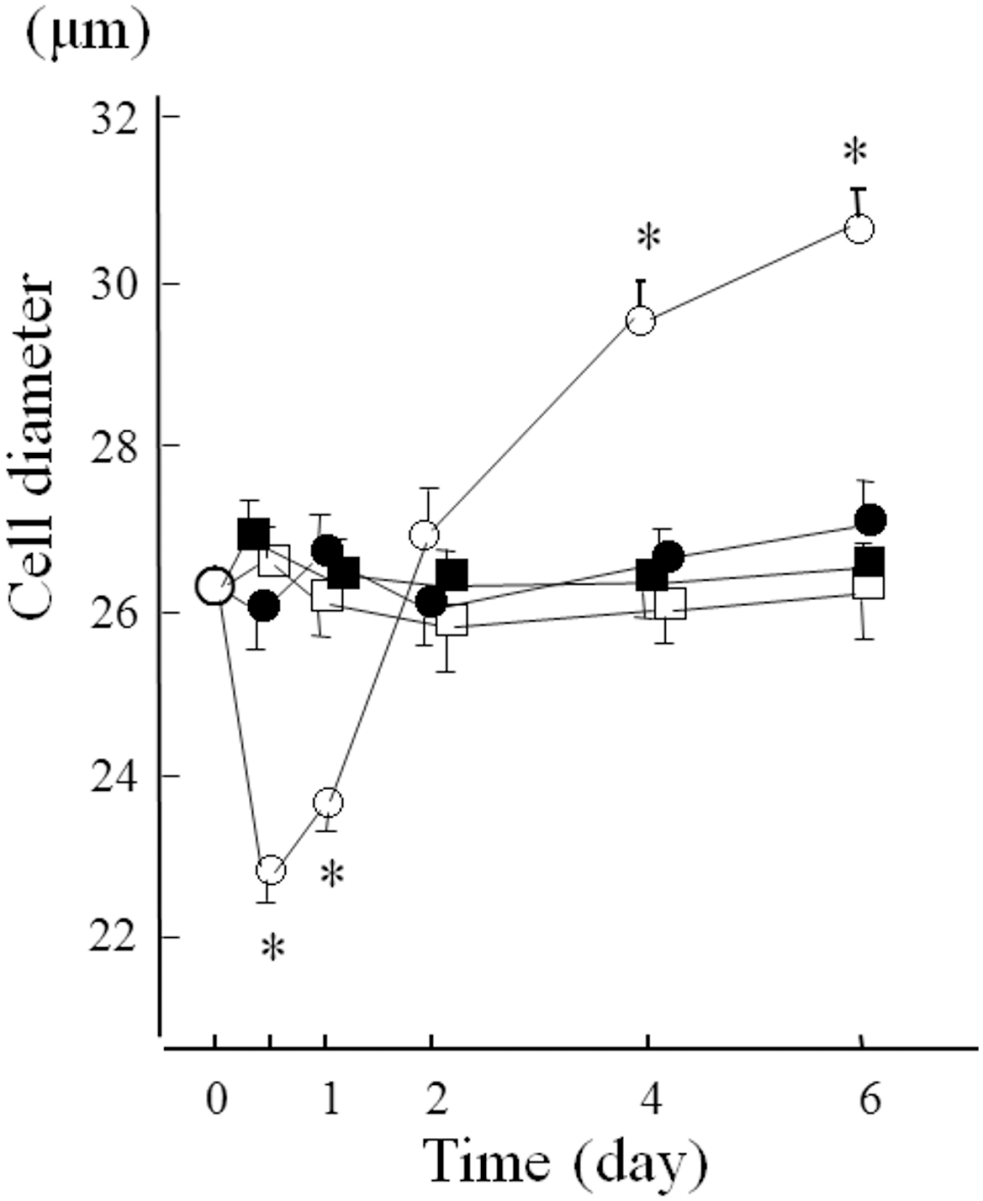
Time-resolved measurements of the cellular size of rat mesangial cells incubated with various conditions for up to 6 days. □: 5 mM glucose, ■: 5 mM glucose with 10^-7^ M phlorizin, ○: 20 mM glucose, ●: 20 mM glucose with 10^-7^ M phlorizin. Determination of cellular size was repeated in different rat mesangial cell preparations, measuring more than 100 cells in each condition. The data are the mean ± SD. *: p<0.01 *vs* 5 mM glucose.

In the experiment with the long-term high glucose condition, the cellular size significantly decreased in 20 mM glucose without phlorizin after the first day, increased to the same size under 5 mM glucose after the second day, and increased significantly after the 4th and 6th days. However, there were no significant differences in the cellular sizes among the 5 mM glucose, 5 mM and 20 mM glucose with phlorizin conditions (Fig 7).

**Fig 8:**
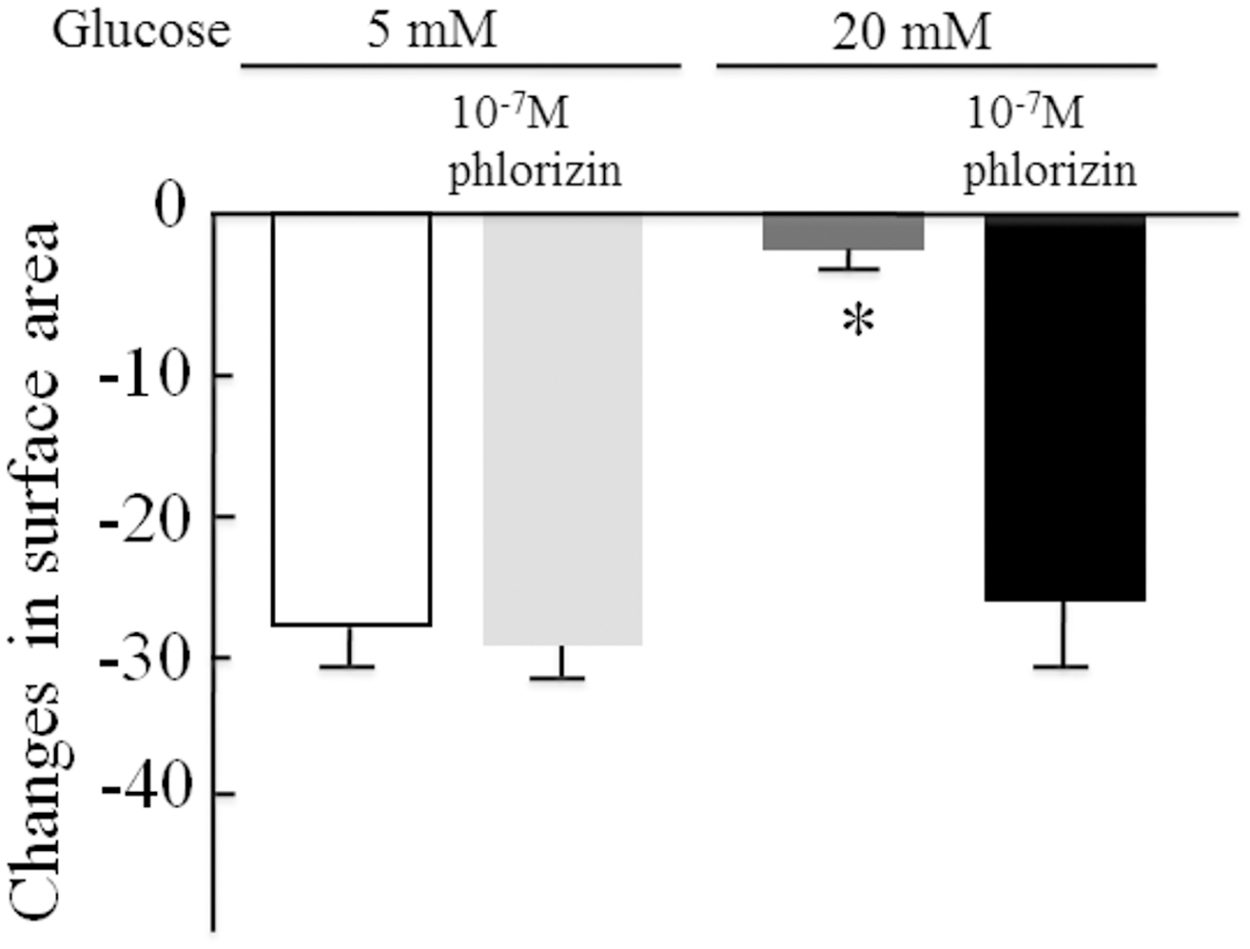
The contractile response to angiotensin II in rat mesangial cells incubated with 5 mM and 20 mM glucose with or without phlorizin for 6 days. n = 6 in each condition. The data are the mean ± SD. *: p<0.01 *vs* 5 mM glucose.

The rat mesangial cells that were incubated for 6 days with 20 mM glucose did not contract in response to angiotensin II; however, the cells that were incubated with 10^-^ M phlorizin in addition to 20 mM glucose did contract in response to angiotensin (Fig 8).

## Discussion

We identified the SGLT isoform in rat mesangial cells by demonstrating sodium-dependent and phlorizin-sensitive glucose uptake [3]. The reported Km and Vmax values of rat mesangial cells were high enough to attribute the SGLT activity in rat mesangial cells to SGLT2. In this study, the IgG antibody developed in our laboratory against SGLT2 yielded a band corresponding to a membrane protein from rat mesangial cells with the predicted molecular weight of 73 kDa, which is identical to the size detected in renal proximal tubular epithelial cells.

Although we repeated the experiments to get a clear single band of SGLT2 in membrane proteins from rat mesangial cells and proximal epithelial cells, the protein bands below 73 kDa were reproducible, and could not be attributed to technical problems. The additional bands are most likely derived from degraded products of membrane proteins produced during many times of ultracentrifugation. Technical differences in getting samples could have resulted in the more pronounced protein bands below 73 kDa in proximal renal tubular cells than in mesangial cells, i.e., samples from rat mesangial cells were obtained from the single layer cultured cells, while renal proximal tubular cells were directly collected by the sieving method. An RT-PCR analysis of SGLT2 in rat mesangial cells also yielded a band of the predicted size, 422 bp, which is identical to the size detected in rat renal proximal tubular epithelial cells. SGLT2 is localized in renal proximal tubular epithelial cells, and this study shows for the first time that SGLT2 is also expressed in rat mesangial cells. SGLT1 was reported to be absent in rat mesangial cells, as assessed by RT-PCR [39].

The properties of SGLT2 and SGLT1, including the Km values for D-glucose and Na^+^ among the human, rabbit, and rat isoforms, have been reported [8]. We reported that the Km value for the D-glucose transporter in rat mesangial cells and bovine retinal pericytes were 1.93 mM [3] and 2.8 mM [4], respectively. Moreover, we reported that bovine retinal endothelial cells, other than retinal pericytes and mesangial cells, do not perform sodium-dependent glucose uptake [4]. The properties and kinetics of the glucose transporter in rat mesangial cells were identical to those of SGLT2. These observations led us to conclude that SGLT2 is expressed in rat mesangial cells.

The cell surface area is reported to correlate with cellular contraction [40]. Our study revealed a glucose concentration-dependent change in mesangial cell surface area, as we reported in bovine retinal pericytes [4]. Moreover, AMG, which enters cells primarily through SGLT [41], induced cellular contraction. In contrast, 2DOG, which enters cells via facilitated glucose transporters [42], could not induce cellular contraction. Therefore, the entry of Na^+^ and glucose through SGLT2 is apparently important for the regulation of glucose-dependent cellular tone, which indicates that in ran mesangial cells, SGLT2 acts as a glucose sensor that regulates the cellular tone.

**Fig 9:**
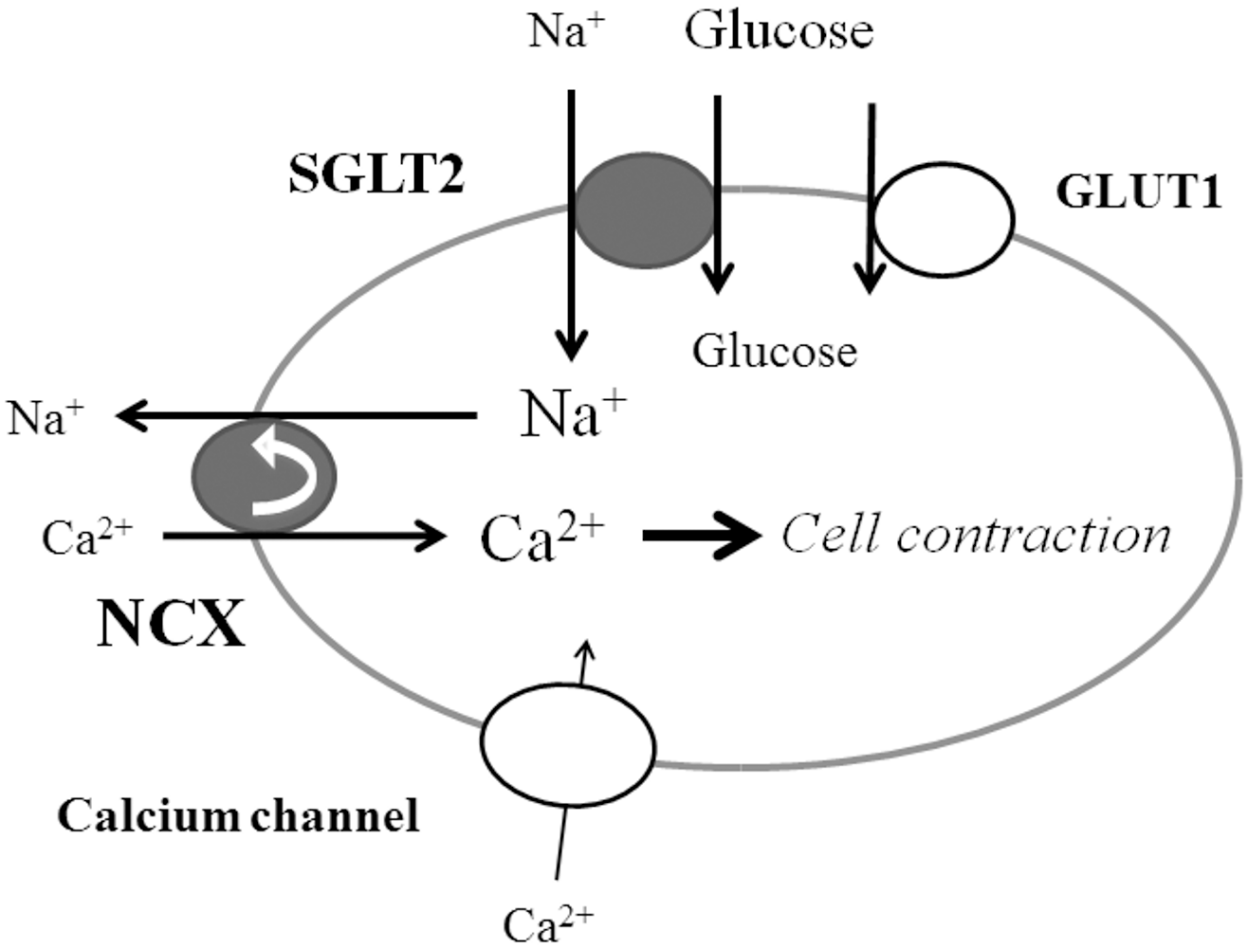
Schematic representation of the effect of SGLT2 on mesangial cells. Glucose enters the cells through both a facilitative glucose transporter (GLUT1) and a sodium-coupled glucose transporter 2 (SGLT2). Na^+^ entry through SGLT2 according to extracellular glucose concentrations brings about Ca^2+^ influx through the sodium-calcium exchanger (NCX), which induces cellular contraction.

The absence of either Na^+^ or Ca^2+^ abolished glucose-induced cellular contraction. Inhibition of SGLT2 by phlorizin and NCX by benzamil-HCl and 2’,4’-dichlorobenzamil-HCl also abolished glucose-induced cellular contraction. Nicardipine, a calcium channel blocker, inhibited angiotensin II-induced cellular contraction, which indicates that the cultured mesangial cells we used have a voltage-dependent calcium channel. However, nicardipine had no effect on glucose-induced cellular contraction. Calcium channel blockers are reported to inhibit NCX; however, the inhibitory effect of these blockers on NCX is reported to be very weak [43]. Glucose-dependent cellular contractility appears to be independent of calcium channels. These observations suggest that the simultaneous entry of sodium with glucose into the cells is essential for glucose-dependent cellular tone and that this regulation depends on both SGLT2 and NCX through the exchange of the intracellular Na^+^ that has entered through SGLT2 with a Ca^2+^ via NCX. Our data indicate that the cellular contractility of mesangial cells depends on calcium entry through both voltage-sensitive calcium channels and NCX, as shown in Fig 9.

A reduction in the size of cultured mesangial cells after exposure to short-term high glucose concentrations has often been reported [44, 45]. Hurst et al. [44] reported a consistent decrease in mesangial cell size, and Derylo et al. [45] also reported cell contraction after exposure to high glucose levels for 60 min. The physiological relevance of the glucose-dependent cellular contractility of mesangial cells is currently unknown. However, acute elevation of blood glucose in healthy volunteers is associated with maintenance of a constant glomerular filtration ratio (GFR) despite an increase in renal blood flow [46]. A novel function of SGLT2 in regulating the contractility of mesangial cells may contribute to the maintenance of a constant GFR under different glucose conditions because the contraction of mesangial cells can regulate glomerular blood flow and filtration [15, 16].

The high glucose-induced cellular contraction was observed for up to 36 hr, and the cellular contraction disappeared after 2 days of the incubation with 20 mM glucose. The cellular size then increased afterwards. This phenomenon seems to mimic the cellular swelling observed in pericytes that swell in the early stage of diabetic retinopathy [22–24]. We reported pericyte swelling 7 days after their placement in a high glucose condition [5]. We hypothesize that the cellular swelling was caused by the intracellular accumulation of Na^+^ through the SGLT2 of bovine retinal pericytes. The pericyte swelling with decreased cellular proliferation was attenuated by incubation with phlorizin [5]. The use of phlorizin blocked glucose entry through SGLT2 in rat mesangial cells and bovine retinal pericytes; thus, another mechanism of cellular swelling is the normalization of intracellular glucose metabolism, such as reversal of the effects of sorbitol and di-acyl-glycerol-protein kinase C, which are reported to suppress Na-K ATPase. We reported that phlorizin attenuated the high glucose-induced changes in the intracellular glucose, sorbitol and fructose levels [5].

Conversely, decreased contraction of mesangial cells has been reported following exposure to long-term high glucose conditions [19, 20]; this decrease is thought to be one of the underlying mechanisms for the hyperfiltration in diabetic nephropathy. We reported that the contractile response to angiotensin II decreases in cells after 5 days of exposure to 20 mM glucose [47]. In this study, the contractile response to angiotensin II in rat mesangial cells was abolished after 6 days incubation with 20 mM glucose. Interestingly, this abolished contractile response was normalized by SGLT2 inhibition with 10^-7^ M phlorizin.

We reported that mesangial cells and pericytes possess facilitated glucose transporter 1 (GLUT1) and SGLT, through which glucose enters at a ratio of almost 1:1 [3]. By inhibiting glucose entry through SGLT2 using phlorizin, we demonstrated that the normalization of excess glucose uptake under high glucose conditions by phlorizin attenuated the overproduction of type IV collagen, which decreased the cellular growth in bovine retinal pericytes [4, 5]. Moreover, we demonstrated glucose-dependent cellular contraction via SGLT2 in this study. The use of SGLT2 inhibitors as anti-diabetic agents that inhibit glucose and Na^+^ entry through SGLT2 in mesangial cells may inhibit excess glucose entry under high glucose conditions; thus, these inhibitors may also normalize cellular swelling and abnormal intracellular glucose metabolism, such as the changes in the polyol pathway and PKC activation under high glucose conditions in mesangial cells and pericytes, independently of the extracellular glucose concentration.

Recently, SGLT2 inhibitors have been used as potent oral hypoglycemic agents [10]. SGLT2 inhibitors were reported to ameliorate diabetic nephropathy, such as by attenuating urinary albumin excretion [48]. Terami et al. reported improved hyperglycemia in response to dapagliflozin, which ameliorated diabetic nephropathy symptoms, such as albuminuria and the accumulation of type IV collagen, independently of glycemic control [49]. This study showed that rat mesangial cells express SGLT2, and we have previously reported identical sugar uptake properties in bovine retinal pericytes and rat mesangial cells; thus, SGLT2 inhibitors may have favorable direct effects on diabetic nephropathy and diabetic retinopathy.

## Conclusion

SGLT2 is present, senses glucose concentrations, and regulates contractile responses of rat mesangial cells via SGLT2 and NCX. These function of SGLT2 may have physiological and pathological relevance in rat mesangial cells.
